# Scopolin Prevents Adipocyte Differentiation in 3T3-L1 Preadipocytes and Weight Gain in an Ovariectomy-Induced Obese Mouse Model

**DOI:** 10.3390/ijms21228699

**Published:** 2020-11-18

**Authors:** Eunkuk Park, Chang Gun Lee, Jeonghyun Kim, Eunguk Lim, Subin Yeo, Seon-Yong Jeong

**Affiliations:** 1 Department of Medical Genetics, Ajou University School of Medicine, Suwon 16499, Korea; jude0815@hotmail.com (E.P.); dangsunsang@naver.com (C.G.L.); danbi37kjh@hanmail.net (J.K.); eunguk@ajou.ac.kr (E.L.); 2 Department of Biomedical Sciences, Ajou University Graduate School of Medicine, Suwon 16499, Korea; 3 Nine B Company, Daejeon 34121, Korea; snsnans@naver.com

**Keywords:** obesity, scopolin, adipogenesis, OVX-induced obese mice, anti-obesity effect

## Abstract

Obesity is prevalent in modern human societies. We examined the anti-obesity effects of scopolin on adipocyte differentiation in preadipocyte 3T3-L1 cells and weight loss in an ovariectomy (OVX)-induced obese mouse model. Scopolin inhibited adipocyte differentiation and lipid accumulation in the preadipocyte cells by suppressing the transcription of adipogenic-related factors, including adiponectin (*Adipoq*), peroxisome proliferator-activated receptor gamma (*Pparg*), lipoprotein lipase (*Lpl*), perilipin1 (*Plin1*), fatty acid-binding protein 4 (*Fabp4*), glucose transporter type 4 (*Slc2a4*), and CCAAT/enhancer-binding protein alpha (*Cebpa*). In OVX-induced obese mice, administration of scopolin promoted the reduction of body weight, total fat percentage, liver steatosis, and adipose cell size. In addition, the scopolin-treated OVX mice showed decreased serum levels of leptin and insulin. Taken together, these findings suggest that the use of scopolin prevented adipocyte differentiation and weight gain in vitro and in vivo, indicating that scopolin may be a potential bioactive compound for the treatment and prevention of obesity in humans.

## 1. Introduction

Obesity is a common metabolic health problem in modern societies [1]. Obesity is associated with an expansion in the size and number of adipocytes, which disrupts energy homeostasis and results in expanded total cholesterol and triacylglycerol (TG) accumulation in the body [2]. Excessive lipid accumulation by adipose tissue is also linked to other metabolic disorders, such as type 2 diabetes, hypertension, and cardiovascular disease [3,4,5]. In addition, the differentiation and maturation of adipocytes plays an important role in the induction of obesity-related diseases [6]. Adipocyte differentiation is regulated by several adipogenic transcription factors, including CCAAT/enhancer-binding proteins (C/EBPs) and peroxisome proliferator-activated receptor gamma which synergistically activate the transcription of downstream target genes such as perilipin 1(Plin 1), fatty acid-binding protein 4, and fatty acid synthase (FAS), leading to fatty acid storage, glucose metabolism, and lipid vacuole formation [7,8]. Lipoprotein lipase in adipocytes is activated by insulin and is involved in the lipolysis of circulating lipoproteins for the subsequent uptake by adipocytes [9].

The most common interventions for obesity are eating a healthy diet and exercising in order to lose weight. However, pharmacological drugs for decreasing food intake or reducing fat accumulation may be considered for obese patients [4]. While various medications are available for the effective treatment of obesity, these medications have numerous adverse effects, especially with long-term treatment. Alternatively, ethnopharmaceutical formulations derived from plants have been widely used to prevent the development of obesity with fewer side effects than conventional pharmaceuticals, and are suitable for long-term use [10,11,12,13,14]. The *Lycii radicis* cortex (LRC) is used as a traditional medicine in East Asia owing to its beneficial effects on bone loss, rheumatoid arthritis, inflammation, and type 2 diabetes [15,16,17]. Phytochemical studies have shown that the LRC consists of various bioactive compounds that contribute to multifactorial positive effects in various diseases [18]. Previous studies have demonstrated that single bioactive components isolated from natural products have therapeutic effects. Scopolin is a bioactive compound derived from the LRC and demonstrates protective effects against inflammation, osteoarthritis, and Alzheimer’s disease [19,20]. Despite the numerous beneficial effects of scopolin, its inhibitory effects on obesity have not been studied.

The aim of the present study was to evaluate the anti-obesity effects of scopolin under in vitro and in vivo conditions. We isolated and identified scopolin from the LRC as a primary bioactive compound. We investigated the effects of scopolin on adipogenesis in preadipocyte 3T3-L1 cells and weight loss in an ovariectomy (OVX)-induced obese mouse model.

## 2. Results

### 2.1. Scopolin Suppresses Adipocyte Differentiation in 3T3-L1 Cells

**Figure 1:**
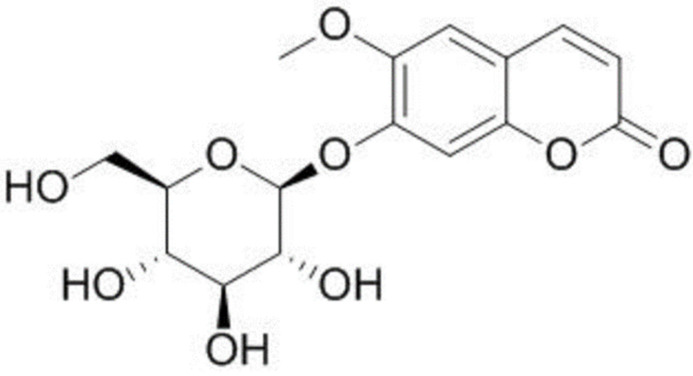
Chemical structure of scopolin.

**Figure 2:**
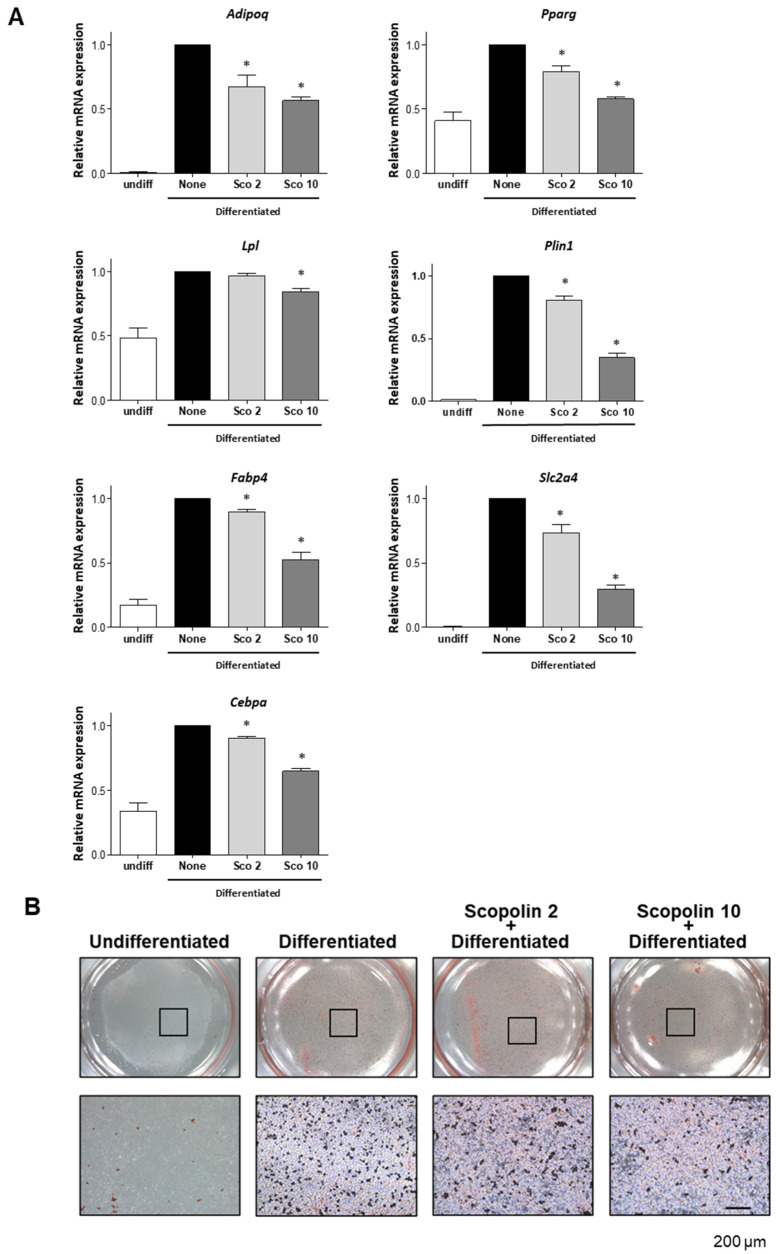
Scopolin inhibited adipocyte differentiation under in vitro conditions. 3T3-L1 preadipocyte cells were differentiated into adipocytes and treated with different concentrations of scopolin (2 or 10 μM) during lipid accumulation. (**A**) The mRNA expression levels of adipogenic markers were assessed by qRT-PCR using targeted gene-specific primers. The relative gene expressions were normalized by mouse glyceraldehyde 3-phosphate dehydrogenase (*Gapdh*) gene. * *p* < 0.05 vs. none (Tukey’s honest significant difference (HSD) post-hoc test and ANOVA). (**B**) Intracellular lipid droplets of differentiated adipocytes were stained with oil red O. Abbreviations: Undiff, undifferentiated; None, non-treated.

To investigate the anti-obesity effects of the bioactive compound scopolin (Figure 1), we first examined whether scopolin inhibited adipocyte differentiation under in vitro conditions. Adipogenic differentiation was induced in mouse preadipocyte 3T3-L1 cells with 3-isobutyl-1-methylxanthine, dexamethasone, and insulin (MDI) in the presence or absence of scopolin (2 and 10 μM). Adipocyte differentiation was examined by quantitative reverse-transcription PCR (qRT-PCR) and oil red O staining. MDI-induced adipogenic differentiation of the 3T3-L1 cells was accompanied by an increase in the mRNA expression levels of adiponectin (*Adipoq*), *Pparg*, (*Lpl*), *Plin1*, *Fabp4*, glucose transporter type 4 (*Slc2a4*), CCAAT/enhancer-binding protein alpha (*Cebpa*), and cells that stained positively for oil red O. However, scopolin treatment decreased the expression of adipogenic transcription factors (Figure 2A) and the number of oil red O positive cells in a dose-dependent manner (Figure 2B). In addition, scopolin did not affect cellular proliferation in the 3T3-L1 cells under any of the experimental conditions used in this study (Figure S1). These results indicate that scopolin prevented adigenic differentiation and lipid accumulation in preadipocytes by inhibiting the transcript expression of adipogenic genes.

### 2.2. Scopolin Reduces Body Weight in OVX Mice

**Figure 3:**
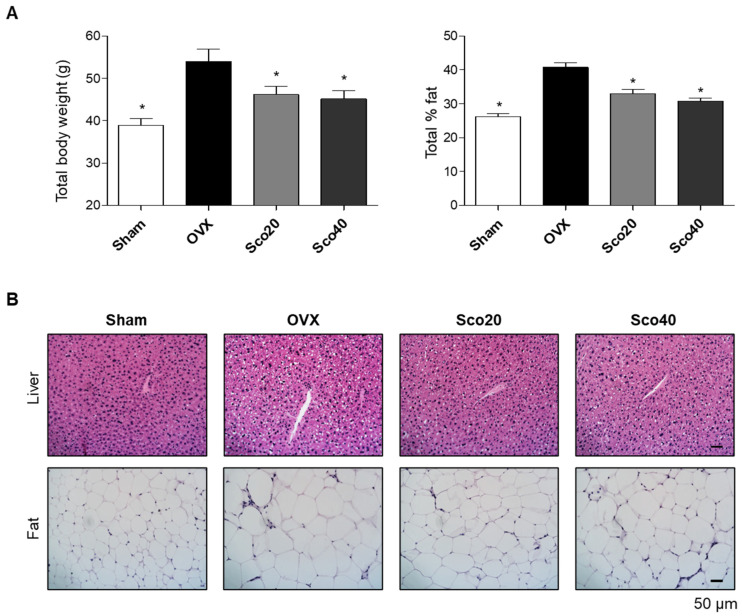
Scopolin inhibited an OVX-induced increase in body weight, hepatic steatosis, and the size of the adipocytes in mice. (**A**) Total body weight and body fat percentage after 12 weeks of non-scopolin administration (Sham and OVX) or scopolin administration (Sco20 and Sco40 mg/kg/day). * *p* < 0.05 vs. OVX (Tukey’s HSD post-hoc test and ANOVA). (**B**) Hematoxylin and eosin (H&E) images of mouse liver and fat tissue were visualized using an optical microscope. Abbreviations: Sham, sham-operated mice; OVX, ovariectomized mice; Sco20 and Sco40, scopolin administration of 20 and 40 mg/kg/day, respectively.

Based on our in vitro results, we examined the anti-obesity effects of scopolin in vivo using an OVX-induced obese mouse model. The OVX or sham-operated (Sham) eight-week-old ddY female mice were divided into four groups, and two different concentrations of scopolin were administered for 12 weeks: (1) Sham, (2) OVX, (3) OVX administered with scopolin 20 mg/kg/day (Sco20), and (4) OVX administered with scopolin 40 mg/kg/day (Sco40). Food intake did not differ among the groups, and the body weight and body fat percentage were analyzed at 0, 6, and 12 weeks (Figures S2 and S3). As expected, the OVX mice demonstrated a significant elevation in total body weight and total body fat percentage. However, treatment with scopolin resulted in a decrease in total body weight and total body fat percentage compared to the OVX mice group at 12 weeks (Figure 3A). In addition, histological images of the liver and fat tissues from the 12-week OVX-control groups were characterized by severe cytoplasmic vacuoles in the liver and significant enlargement of adipocytes in the epididymal fat. However, scopolin administration decreased the number of hepatic vacuoles and reduced the adipocyte size in the fat tissue (Figure 3B). These results suggest that scopolin treatment inhibited OVX-induced weight gain by suppressing hepatic fat deposition and enlargement of adipocyte size.

### 2.3. Scopolin Reduces Levels of Obesity-Associated Hormones

**Figure 4:**
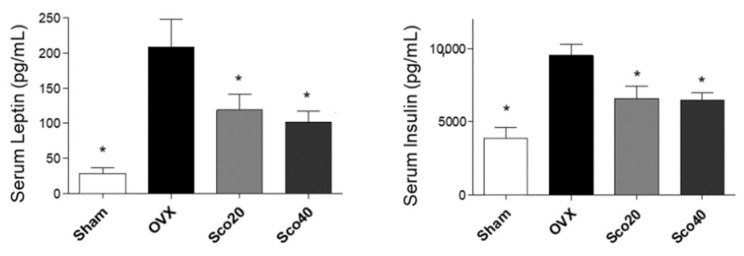
Scopolin decreased serum concentrations of leptin and insulin in OVX-induced obese mice. Blood samples after 12 weeks of non-administration of scopolin (Sham and OVX) and scopolin administration (Sco20 and Sco40 mg/kg/day) were collected and serum levels of leptin and insulin were assessed by ELISA. * *p* < 0.05 vs. OVX (Tukey’s HSD post-hoc test and ANOVA). Abbreviations: Sham, sham-operated mice; OVX, ovariectomized mice.

We further examined the anti-obesity effects of scopolin in vivo by evaluating the serum concentrations of obesity-associated hormones such as leptin and insulin. Blood samples were collected from the mice after 12 weeks and the serum levels of leptin and insulin were assessed by enzyme-linked immunosorbent assay (ELISA). As a result, OVX mice showed increased serum levels of leptin and insulin compared to the Sham group, while the scopolin-administered OVX mice showed decreased serum levels of leptin and insulin compared to the OVX mice group (Figure 4). These results suggest that scopolin inhibited OVX-induced obesity under in vivo conditions by reducing obesity hormones such as leptin and insulin.

## 3. Discussion

In the present study, we demonstrated the anti-obesity effects of a bioactive compound, scopolin, under in vitro and in vivo conditions. We observed that scopolin prevented adipocyte differentiation by reducing the mRNA expression levels of adipogenic factors and lipid accumulation in preadipocyte 3T3-L1 cells. Oral administration of scopolin in an OVX-induced obesity mouse model revealed that scopolin prevented OVX-induced weight gain, an increase in body fat percentage, hepatic steatosis, and an increase in epididymal fat by downregulating serum leptin and insulin levels.

Adipocytes play an important role in lipid storage and metabolism [21]. Typically, increased levels of fatty acids induce the regulation of lipid uptake via secretion of adipokines such as adiponectin and leptin in adipocytes [22]. Secreted adiponectin and leptins reduce fatty acid uptake by increasing fat metabolism and insulin sensitivity [23]. However, excessive fat deposition in obesity leads to an unbalanced adipokine expression in adipocytes, resulting in metabolic syndromes such as insulin/leptin resistance [24]. The differentiation of adipocytes is regulated by several key transcription factors, including *Lpl, Pparg*, *Plin1*, *Fabp4*, *Slc2a4*, and *Cebpa*. *Pparg* stimulates lipid uptake and increases insulin sensitivity by enhancing fatty acid storage in adipocytes [25]. *Plin1* is an essential factor required for lipid droplet accumulation, and *Plin1* deficiency leads to lipid dysregulation in adipose tissue [26]. *Fabp4* regulates leptin secretion and mitochondrial fatty acid oxidation [27]. *Slc2a4* transports circulating glucose to adipocytes and skeletal muscle [28]. *Cebpa* promotes new fat formation and accumulation of lipids in the adipocytes and the liver [29]. These adipogenesis-inducing genes are also upregulated when preadipocyte 3T3-L1 cells differentiate into mature adipocytes, including lipid-rich accumulation in the cytoplasm [30]. In this study, scopolin inhibited MDI-induced adipocyte differentiation by reducing the expression of adipogenesis-inducing genes. The results demonstrated an inhibitory effect of scopolin on adipogenesis.

The OVX animal model leads to obesity in both rats and mice and is a well-known feature of human obesity [31,32]. A number of reports have demonstrated that the OVX obesity model is induced by mimicking the imbalance of lipid metabolism in menopausal or post-menopausal women [33,34,35]. In addition, a deficiency in circulating estradiol levels triggers unregulated hepatic lipid homeostasis and enhances the adipogenic potential of mesenchymal stem cells in adipose tissue [36,37]. Our findings demonstrated that scopolin prevented OVX-induced weight gain by decreasing fat mass, hepatic steatosis, and enlargement of adipose tissue, suggesting that scopolin has a beneficial effect on menopausal or post-menopausal obese women.

The clinical significance of serum leptin and insulin levels in obesity has also been reported in the literature because of the positive correlation with an increase in body weight and fat mass [38,39]. The pancreatic hormone insulin is a major regulator of adipogenesis, which is necessary for glucose metabolism in mice and humans [40]. Leptin secreted from mature adipocytes is involved in the uptake of fatty acids and glucose transport [41]. At the onset of obesity, the excessive levels of these two hormones lead to the impairment of their functions in target tissues, resulting in insulin/leptin resistance [42,43]. Therefore, a number of therapeutic strategies against obesity have focused on reducing insulin/leptin resistance [44,45,46]. Our results showed that the administration of scopolin decreased serum levels of insulin and leptin, indicating the inhibition of OVX-induced obesity by preventing insulin/leptin resistance.

## 4. Materials and Methods

### 4.1. Mouse Preadipocyte Cell Culture and Adipogenic Differentiation

Mouse 3T3-L1 preadipocyte cells were maintained in Dulbecco’s modified Eagle’s medium supplemented with 10% FBS, penicillin (100 U/mL), and streptomycin (100 μg/mL). For adipocyte differentiation, 3T3-L1 cells were cultured with 0.5 mM 3-isobutyl-1-methylxanthine, 1 mM dexamethasone, and 1 μg/mL insulin (MDI) for 3 days, then incubated a further 5 days with 1 μg/mL insulin for lipid accumulation. The medium was changed every 3 days. All cultured cells were maintained in a humidified atmosphere at 37 °C with 5% CO_2_.

### 4.2. Water-Soluble Tetrazolium Salt (WST) Assay

The 3T3-L1 cells were seeded in a 96-well plate and incubated with culture media containing different concentrations of scopolin (2 or 10 μM) for 48 h. Cell viability was determined by WST assay using an EZ-Cytox Cell Viability Assay Kit (Daeil; Seoul, Korea). WST solution (20 μL, 5 mg/mL in phosphate-buffered saline) was added and incubated for 4 h. Absorbance was measured at 450 nm and 655 nm using a microplate reader (Bio-Rad; Hercules, CA, USA).

### 4.3. Quantitative Reverse-Transcription PCR (qRT-PCR)

The primers targeting adipogenic markers were as follows: 5′-TGT TCC TCT TAA TCC TGC CCA-3′ and 5′-CCA ACC TGC ACA AGT TCC CTT-3′ for mouse *Adipoq*, 5′-GGA AGA CCA CTC GCA TTC CTT-3′ and 5′-GTA ATC AGC AAC CAT TGG GTC A-3′ for mouse *Pparg*, 5′-ATG GAT GGA CGG TAA CGG GAA-3′ and 5′-CCC GAT ACA ACC AGT CTA CTA CA-3′ for mouse *Lpl*, 5′-CAA GCA CCT CTG ACA AGG TTC-3′ and 5′-GTT GGC GGC ATA TTC TGC TG-3′ for mouse *Plin1*, 5′-AAG GTG AAG AGC ATC ATA ACC CT-3′ and 5′-TCA CGC CTT TCA TAA CAC ATT CC-3′ for mouse *Fabp4*, 5’-ACA CTG GTC CTA GCT GTA TTC T-3’ and 5’-CCA GCC ACG TTG CAT TGT A-3’ for mouse *Slc2a4,* 5′-GCG GGA ACG CAA CAA CAT C-3′ and 5′-GTC ACT GGT CAA CTC CAG CAC-3′ for mouse *Cebpa*, and 5′-TGA CCA CAG TCC ATG CCA TC-3′ and 5′-GAC GGA CAC ATT GGG GGT AG-3′ for mouse *Gapdh*. Relative gene expression levels were normalized to that of mouse *Gapdh*. The values are expressed as fold changes compared to the control. Fold changes are presented as 2^−ΔΔCt^ (ΔΔCt = ΔCt_control_ − ΔCt_treatment_).

### 4.4. Oil Red O Staining

Cells were fixed in 4% paraformaldehyde for 15 min, washed three times with phosphate buffered salin, and stained with oil red O dye for 2 h. Oil red O positive cells were visualized with an optical microscope (Leica, Wetzlar, Germany), and representative images were taken using an attached camera.

### 4.5. Ovariectomized Murine Model Experiment

Either ovariectomized (OVX, *n* = 24) or sham-operated (sham, *n* = 8) 8-week-old female ddY mice were purchased from Shizuoka Laboratory Center Inc. (Hamamatsu, Japan). Mice were maintained on a diet of Formula-M07 (5.0 g/day) (Feedlab Co., Ltd., Hanam, Korea) and tap water (15 mL/day). All mice were housed individually in clear plastic cages under controlled temperature (23 ± 2 °C), humidity (55 ± 5%), and illumination (12 h light/dark cycle) conditions. Mice in the two treatment groups were administered different concentrations of scopolin (20 and 40 mg/kg/day) for 12 weeks. The animal research protocol was approved by the Animal Care and Use Committee of Ajou University School of Medicine (AMC-133; 2019-01-15), and all experiments were conducted in accordance with the institutional guidelines established by this committee.

### 4.6. Blood Sampling and ELISA

Mice were euthanized before blood and tissue collection. Blood samples were collected from the left ventricle of the heart and the serum was separated by centrifugation at 1200× *g* at 4 °C for 15 min. The samples were stored at −20 °C until further analysis. The serum levels of leptin and insulin were assessed using a customized MILLIPLEX^®^ Multiplex Assay for Luminex^®^ according to the manufacturer’s instructions (Merck Millipore, MA, USA).

### 4.7. Histology

Fat and liver tissues were fixed in cold 4% paraformaldehyde in 0.1 M phosphate buffer at pH 7.4. The samples were embedded in paraffin and cut into 3 μm coronal planes using a rotary microtome. Tissue sections were stained with hematoxylin and eosin (H&E) to evaluate histological changes in fat and liver tissues. The size of adipocytes in the fat tissue was evaluated using CaseViewer 2.1 version (3DHSTECH Ltd., Budapest, Hungary) and ImageJ 1.52r version (NIH, Bethesda, Rockville, ML, USA) software.

### 4.8. Statistical Analysis

All experiments were performed in triplicate, and the data are presented as mean ± standard error of mean (SEM). All statistical analyses were performed using the software package SPSS v.11.0 for Windows (SPSS Inc., Chicago, IL, USA). Comparisons of multiple groups were evaluated with a one-way analysis of variance (ANOVA), followed by Tukey’s HSD (honest significant difference) post-hoc test. A probability value less than 0.05 (*p* < 0.05) was considered statistically significant.

## 5. Conclusions 

We demonstrated that scopolin inhibited adipocyte differentiation and lipid accumulation in preadipocyte cells and prevented OVX-induced weight gain in an obese mouse model. Our results suggest that scopolin may be an attractive therapeutic natural medicine for the prevention and treatment of obesity.

## Supplementary Materials

The following are available online: https://www.mdpi.com/1422-0067/21/22/8699/s1.
