# Supplementary material for: Scopolin Prevents Adipocyte Differentiation in 3T3-L1 Preadipocytes and Weight Gain in an Ovariectomy-Induced Obese Mouse Model
Source: Int J Mol Sci. 2020 Nov 18;21(22):8699. doi: 10.3390/ijms21228699 (PMC7698923; doi:10.3390/ijms21228699)
Supplement: Supplementary file 1 [file ijms-21-08699-s001.pdf]

Scopolin attenuates adipocyte differentiation in 3T3-L1 preadipocytes and weight gain in ovariectomy-induced obesity mice.

Eunkuk Park, Chang Gun Lee, Jeonghyun Kim, Eunkug Lim and Subin Yeo & Seon-Yong Jeong

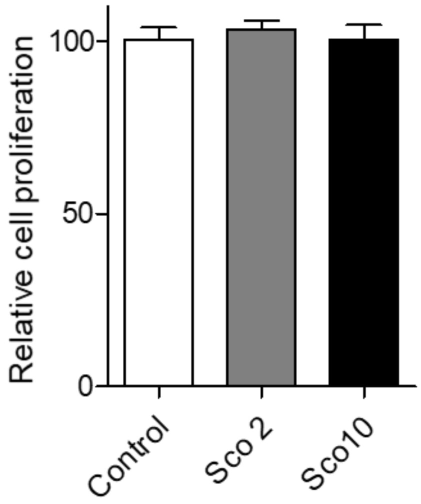

Supplementary figure S1. Effect of scopolin on cell viability in 3T3-L1 cells. The cells were cultured with two different concentrations of Scopolin (2 and 10 μM), and the cell viability were analyzed. .

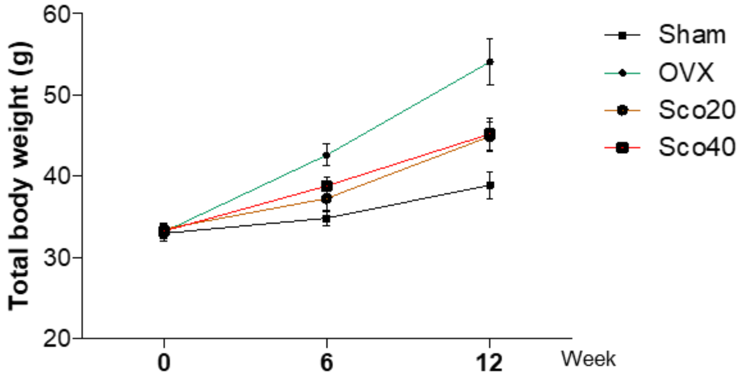

Supplementary figure S2. Changes of total body weight at 0, 6, and 12 weeks

.

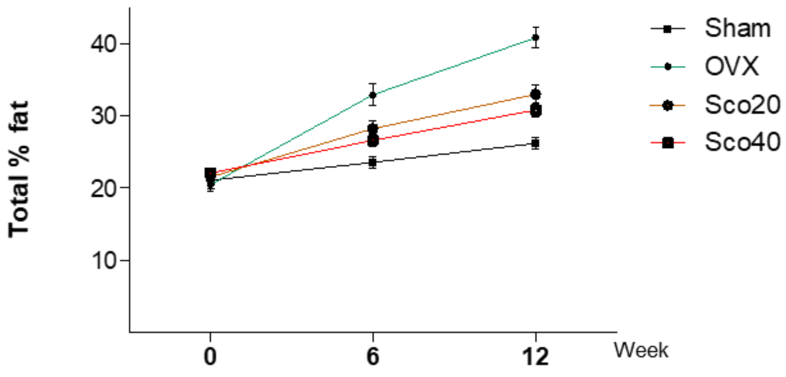

Supplementary figure S3. Changes of total % fat at 0, 6, and 12 weeks.
